# Comprehensive understanding of risk and protective factors related to adolescent pregnancy in low- and middle-income countries: A systematic review

**DOI:** 10.1016/j.adolescence.2018.10.007

**Published:** 2018-12

**Authors:** Hye Won Chung, Eun Mee Kim, Ji-Eun Lee

**Affiliations:** a Department of Obstetrics and Gynecology, School of Medicine, Ewha Womans University, Seoul, South Korea; b Department of International Studies, Graduate School of International Studies, Ewha Womans University, Seoul, South Korea

**Keywords:** Low and middle-income countries, Systematic review, Adolescent pregnancy, Adolescent birth, Risk and protective factors

## Abstract

**Introduction:**

Adolescent pregnancy causes serious problems not only for girls, but also for their family, and society. This study aimed to understand factors related to adolescent pregnancy in low- and middle-income countries using a multilevel approach adopted by Bronfenbrenner's ecological model.

**Methods:**

A total of 11,933 studies published in between 2000 and 2015 were identified in 4 databases. Based on inclusion criteria and risk of bias assessment, a total of 67 articles were retrieved for analysis.

**Results:**

Thematic analysis revealed that early marriage, sexual risk behaviors, substance use, family experience of adolescent birth, peer pressure, and lack of sex education and health service increased the hazards of adolescent pregnancy. Communication with parents, school activities, community meetings, laws, and government policies protected adolescents from pregnancy.

**Conclusions:**

Results of this study suggests that the background of adolescents and complex interactions among various factors should be considered for pregnancy. In future research, mixed-method that supplements the methodological weaknesses of previous studies is also recommended.

## 1 Introduction

An adolescent pregnancy threatens girls' health and restricts their socioeconomic opportunities. Girls have suffered from severe health problems and even died mainly due to maternal conditions such as pregnancy and childbearing (Patton et al., 2009). According to WHO (2015, p. 72), “Globally, in 2012, 37% of YLLs (Years of Life Lost) was due to deaths from maternal causes or deaths among those younger than 15 years”. Today, about 11% of world's total births still come from girls aged from 12 to 15 years (WHO, 2015). These girls are more vulnerable to risks of pregnancy and birth complications than adult women because not only their gynecological condition is not ready, their pelvic growth is not completed either (Vogel et al., 2015). In 42 developing countries, about 2.5 million girls under age 16 gave births every year, and 50% of them were under age of 15 years (Neal et al., 2012; UN, 2009).

Many scholars are aware of the problem of adolescent pregnancy. They have conducted studies on factors and predictors associated with teenage pregnancy. However, up to date, only a few studies have conducted a systematic review to find comprehensive factors related to adolescent pregnancy in low- and middle-income countries. Imamura et al. (2007) have analyzed factors associated with teenage pregnancy through systematic review, but they only examined EU member states. Mmari and Blum (2009) have conducted a structured literature review on factors affecting adolescent reproductive health in developing countries. They limited outcomes as ever had premarital sex, condom use, pregnancy and early childbearing, and sexually transmitted infections and HIV. Although pregnancy and childbearing were considered as outcomes, they did not conduct a thorough review of earlier studies. A recent study conducted by Pradhan, Wynter, and Fisher (2015) is a systematic review similar to this study. They analyzed the literature focusing on factors related to adolescent pregnancy in developing countries. However, they only covered a small number of articles because they used limited search terms, which narrowed the range of studies.

The objective of this systematic review was to provide a comprehensive view of factors associated with adolescent pregnancy in low- and middle-income countries by synthesizing as many related studies as possible. We used a multilevel approach to find comprehensive associations between various factors and teenage pregnancy.

### 1.1 Multilevel approach to adolescent pregnancy

**Fig. 1:**
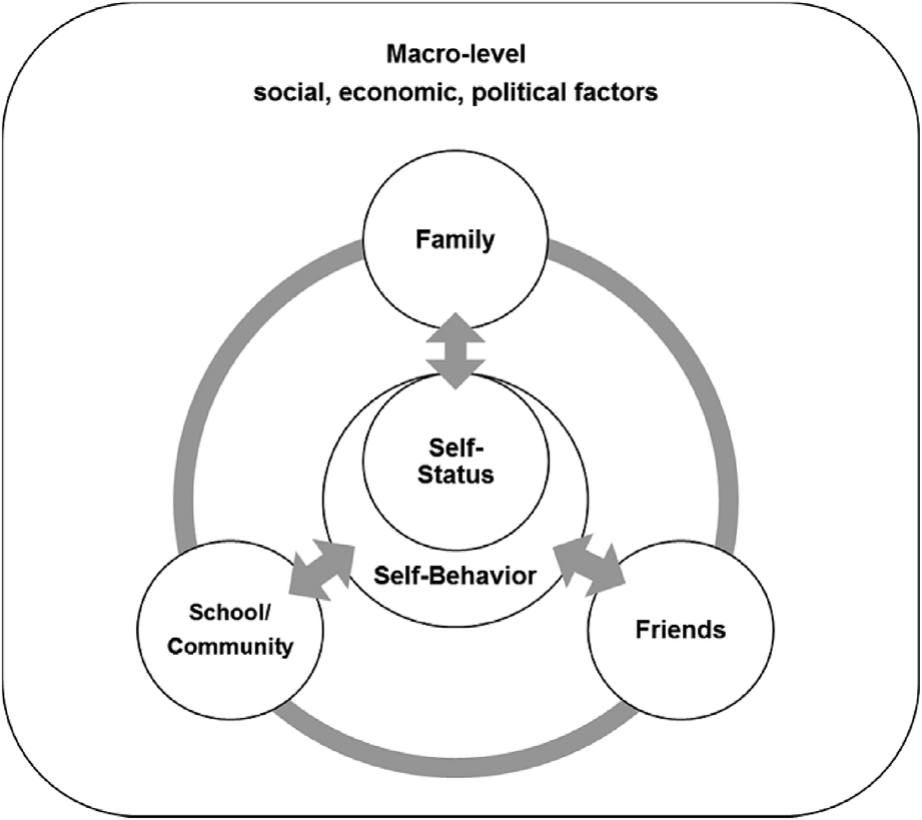
Multilevel approach to adolescent pregnancy.

In the process of physical, mental, and psychosocial development during adolescence, various factors and circumstances can affect an adolescent's attitude, behavior, and life. Bronfenbrenner (1994) has introduced an ecological model of human development considering that human and the environment interact with each other and each is affected by the other. By adopting this model, we developed a framework of a multilevel approach to gain a comprehensive understanding of adolescent pregnancy (Fig. 1). This framework shows that various micro- and macro-level factors such as self-status, behavior, family, friends, school, community and macro-level socio-economic, and political factors are related to adolescent pregnancy. Each factor is directly related to teenage pregnancy. These factors also interact with each other and indirectly affect pregnancy. By using this approach, we analyzed factors that previous studies discovered and how these were related to teenage pregnancy.

## 2 Methods

This study was guided by the PRISMA checklist (Moher, Liberati, Tetzlaff, Altman, & Group, 2009). The following four electronic databases widely used in medical science and social science were searched: POPLINE, PubMed, Web of Science, and PsycINFO. Initial search criteria were scholarly articles or dissertations written in English and published between 2000 and 2015. Search terms were chosen by reviewing references of literature regarding adolescent pregnancy and childbearing to include as many related studies as possible (Table 1). ‘Developing countries’ and ‘low- and middle-income countries’ were not included as search terms since they could exclude studies of a country without mentioning its low- or middle-income status. Therefore, all related studies were searched regardless of country. In order to select articles eligible for our systematic review, the inclusion criteria were used such as country of study site was classified as low- and middle-income country; study aimed to find factors related to adolescent pregnancy or childbearing; and study group included adolescents aged between 10 and 19 years (Table 2). Based on these criteria, titles and abstracts were screened. Finally, selected articles were reviewed and analyzed by organizing information in an Excel database. Risk of bias assessment was conducted using various tools depending on the study design. We used assessment tools including the Joanna Briggs Institute Critical Appraisal Checklist (2016) for analytical cross-sectional studies, National Institutes of Health Quality Assessment Tool (2014) for cohort studies, and Critical Appraisal Skills Programme checklist (2013) for qualitative case-control, cross-sectional survey, and descriptive study. Each tool has different assessment criteria, but most of the tools commonly consider following standards: a clear statement of research question and objective; specified and defined study subject; appropriate methodology and research design used, identified confounding factor, rigorous data analysis, and clear statement of findings. A thematic analysis was conducted to synthesize studies and compare outcomes by categorizing factors through the framework of multilevel approach.

**Table 1:** Search terms.

Column 1	Column 2 (Individually combined with)
Title: (adolescent* OR teen* OR “young maternal” OR “young women” OR “young mother*” OR early OR girl* OR “young people”)	Title: (pregnant* OR childbearing* OR birth* OR childbirth* OR delivery OR deliveries OR mortality OR morbidity OR fertility OR “reproductive health” OR mother* OR marriage OR *married)

Note: We used quotation marks (“ ”) to find the exact words or phase.

**Table 2:** Inclusion criteria.

Criterion	Inclusion
Year	2000–2015
Language	English
Geographic area	Low- and middle- income countries (based on the classification of the World Bank)
Outcome measured	Factors related to adolescent pregnancy or childbearing
Study population	Study group includes adolescents aged 10-19
Study type	Qualitative, quantitative or mixed method

## 3 Results

**Fig. 2:**
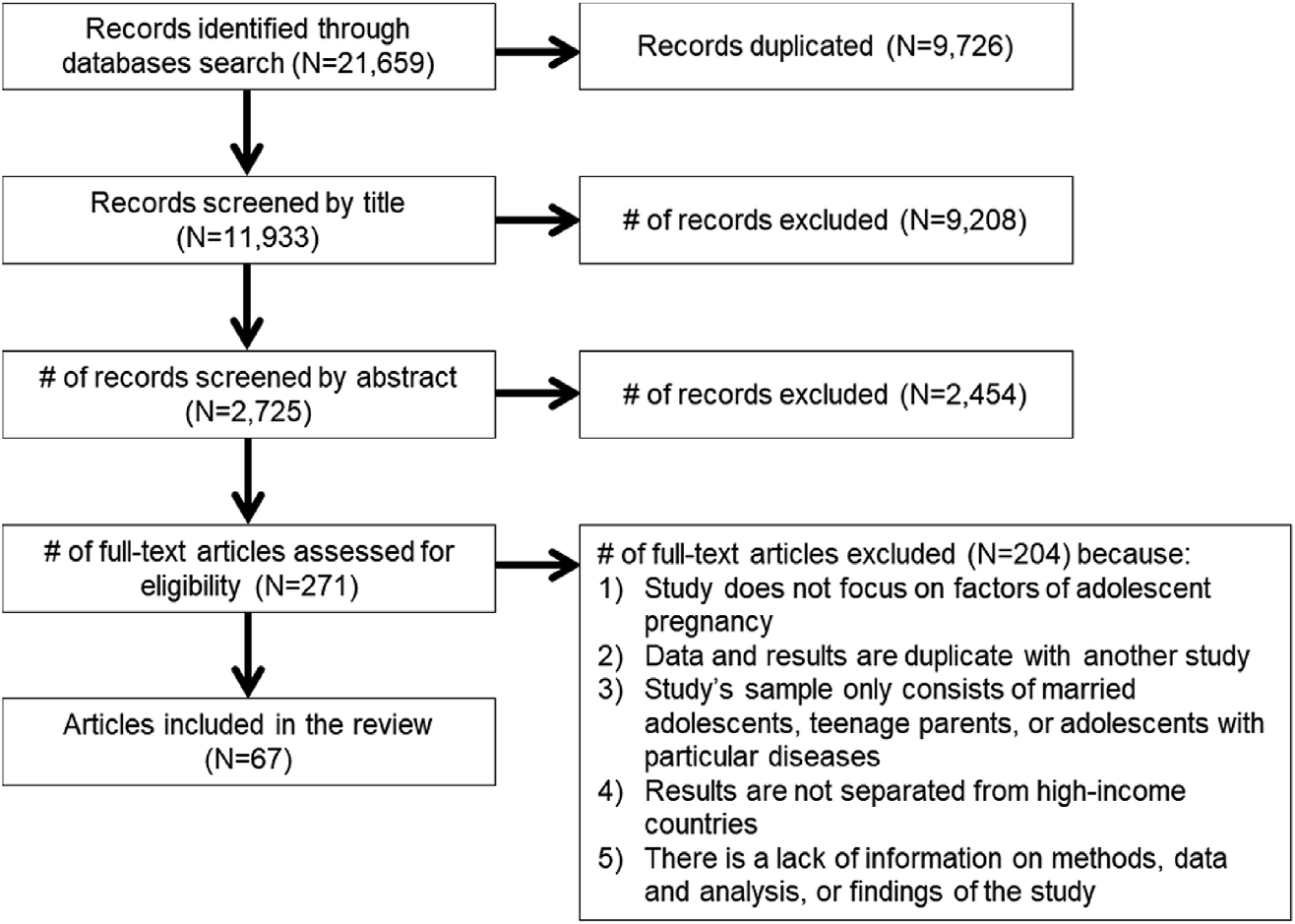
Flow of search process.

In the first stage, 21,659 articles were retrieved, including duplicates from four databases: Web of Science (n = 9319), PubMed (n = 7353), PsycINFO including dissertations (n = 3460), and POPLINE (n = 1527) (Fig. 2). In the second stage, 11,933 articles were screened by titles after excluding duplicates. A total of 9208 articles were excluded because they were not related to adolescent pregnancy. In the third stage, of 2725 remaining articles, 2454 studies were excluded by abstract screening referring to the inclusion criteria. In the fourth stage, full-text papers of the remaining 271 articles were reviewed, and 204 articles were excluded due to one of the following reasons: 1) the study did not focus on factors of teenage pregnancy; 2) the study's data and results were duplicates of another study; 3) the study's samples only consist of married adolescents, teenage mothers, or adolescents with a particular health condition such as having HIV or STIs; 4) results were not separated from high-income countries; and 5) there was a lack of information on its methods, data, and analysis, or findings of the study. Finally, a total of 67 articles were selected for our analysis.

### 3.1 Characteristics of included studies

The 67 studies included in our analysis were from many different low- and middle-income countries, including seven countries in Asia, eleven in Africa, nine in Latin America, one in Europe, and several developing countries as a group. These studies used various research methods, including thirty-five analytical cross-sectional studies, five qualitative studies, seven case-control studies, three cohort studies, two randomized controlled trials, one panel study, nine cross-sectional surveys, three descriptive studies, and two mixed method studies. Details of methods used in these studies are presented in Appendix.

### 3.2 Factors associated with adolescent pregnancy using multilevel approach

A thematic analysis was used to present various risk and protective factors for adolescent pregnancy. The factors are categorized according to multilevel approach: Self-status, self-behavior, family, friends, school/community, and macro-level social, economic, and political factors (Table 3).

**Table 3:** Risk and protective factors for adolescent pregnancy.

Theme	Factors
**Risk factors**	
Self-status	Older age, low self-esteem, high internal locus of control, unemployment, working as a housemaid, low socioeconomic class, poverty, household shock, unmarried, married before 15, living in rural or other urban area outside of capital city, rural birthplace, race, ethnicity, not being religious, no religion (vs. Catholics), migrant status, not being happy as a teen, having strong gender bias, having higher sexually permissive attitudes, concerns about the side-effects of contraceptive methods, believing that contraception is a woman's responsibility
Self-behavior	Substance use, younger age at first intercourse, non-use of contraception during first sex, frequent sex without injectable contraceptives, experience of sexual abuse, physical abuse, forced sexual initiation, experience of pregnancy, ever engaging in transactional sex, low or average level of contraceptive knowledge, difficult to obtain accurate information on reproduction and contraception, unwilling to buy contraceptives at the centers or pharmacy, poor sexual negotiation skills
Family	Absence of both parents in some periods of adolescence, not living with a father, being raised by single parent, living in a mother-headed household (vs. father-headed), mother gave a birth during adolescence, having an employed mother, improper care from the family or lack of parental care, authoritarian parenting, permissive parenting style, lack of communication between parents and children, maternal religious belief (traditional), no sex education at home, parental influences to marry early, having taken care of other children, sibling with history of adolescent pregnancy, more than 3 siblings, had siblings of different fathers, living in a large household
Friends/Partner	Currently in a stable relationship with a boyfriend, first sexual partner was 5 years older, partner was a similar age, discuss sexual matters or pregnancy with boyfriends, beliefs that most friends are pregnant, increasing number of lifetime partners, having girlfriends with health-risk behaviors, partner or peer pressure to have sex and not to use contraceptives, get sex information from peers, sexual coercion by boyfriend, intimate partner abuse, boyfriend's refusal to use condom
School	School dropout, long-term school absences, low level of education, no education, unsupervised activities after school, less participated extra activities at school, lack of sex education from schools, poor quality of sex education, going public school, being students (vs. completed education)
Community	Neighborhood violence, fear of being robbed or attacked, not having access to health service, church forums that discuss sex/family life, socio-cultural modernization (e.g., attending schools away from home), lack of entertainment and social infrastructure
Macro-level factors	Income inequality, fall of socialist regime, low population density, low human development index, percent of families supported by the social assistance program, low Municipal development index, low social responsibility index, government's safe sex programs did not target adolescents
**Protective Factors**	
Self-status	Being young, being involved in community groups, higher levels of family income, lower family income, being married, unmarried, religion, Christianity (vs. Islam), affiliation with Islam, traditional religion, Protestants (vs. Catholics), Pentecostal affiliation, attend worship services frequently, having a white collar job, non-agricultural worker, living in more developed district, urban residence, rural residence, ethnicity
Self-behavior	Experience sexual violence, think important to protect self against pregnancy, using a condom at first sex, consistent condom use
Family	Higher levels of maternal schooling and paternal schooling, father is present in the household, parents are affectionate, parents are supportive, good parental care and counseling
Friend/Partner	Discuss sexual matters with peers, get together with friends often
School	Being in school, school term (vs. vacation), female secondary enrollment
Community	Better perceived physical environment, youth forums on sex education
Macro-level factors	Strict laws setting the minimum age of marriage at 18, population reduction policies, international treaties, consistent law, education subsidy program, joint program, free education by government

Note: This table shows the list of factors for adolescent pregnancy that are presented from 67 included studies. Several factors have a comparison that is in the parenthesis. For example, no religion (vs. Catholics) as a risk factor means that adolescents who have no religion are more likely to get pregnant than those who are Catholics.

#### 3.2.1 Self-status

Self-status refers to a person's profile that directly tells his/her current situation such as current age, education, religion, income, and employment status. Many scholars have found a co-relationship between the teenager's age and pregnancy. By using multivariate statistical analyses, they have argued that age is positively associated with adolescent pregnancy (e.g., Alemayehu, Haider, & Habte, 2010; Calvert et al., 2013). Cross-sectional survey in Ghana and South Africa have shown similar findings that pregnant girls are older than non-pregnant girls (Agyei, Biritwum, Ashitey, & Hill, 2000). However, when it comes to unwanted or unintended pregnancy, younger adolescents have shown a higher likelihood of being pregnant than older adolescents or adults (e.g., Amoran, 2012).

Education is the most commonly associated factor in many studies. They have shown that a higher level of educational attainment or more schooling is a protective factor against pregnancy (e.g., Gupta & Mahy, 2003; Sahoo, 2011). Adolescents who have no formal education or lower level of education, or who are not enrolled in school have a higher likelihood of being pregnant (Gomes, Speizer, Gomes, Oliveira, & Moura, 2008; Omar et al., 2010). Long-term school absences, temporary dropout of school, and school failure are also risk factors for pregnancy (e.g., Almeida, Aquino, & Barros, 2006; Gigante, Victora, Gonçalves, Lima, & Barros, 2004). Being a student has been found to be a protective factor against pregnancy in several studies (e.g., Beguy, Mumah, & Gottschalk, 2014). However, another study has found that being a student makes them vulnerable to pregnancy compared to adolescents with a decent job (Amoran, 2012). It has also been reported that adolescents who attend public schools are more likely to be pregnant than those who attend private schools (Moron-Duarte, Latorre, & Rafael Tovar, 2014). The possible reason for this result would be more disadvantaged students at a public school than private school due to the higher cost of private school tuition than that of public school.

Also, the studies have shown a significant co-relationship between economic status and adolescent pregnancy. Adolescents with low economic status, poverty, and smaller income are more likely to become pregnant than their counterparts (Maswikwa, Richter, Kaufman, & Nandi, 2015; McHunu, Peltzer, Tutshana, & Seutlwadi, 2012). Income inequality is also a risk factor for adolescent pregnancy (Chiavegatto Filho & Kawachi, 2015). However, some studies have shown no association between income and pregnancy. For example, Marteleto, Lam, and Ranchhod (2008) have studied 3916 young people in South Africa and found that income is not significantly related to pregnancy.

Several studies have shown that marital status is significantly associated with pregnancy during adolescence. Some studies found that married girls or women who were married in adolescence are more likely to be pregnant than those who were not married in their adolescence (Beyene et al., 2015; Gurmu & Dejene, 2012). On the other hand, Omar et al. (2010) have shown that there are more unmarried adolescents in the case group—pregnant girls—than those in the control group—women who were not pregnant in adolescence. However, their study did not include an age-matched control group for the case group, which might have the risk of selection bias. Other study has focused on unplanned pregnancy and found that not being married is positively associated with adolescent pregnancy (Calvert et al., 2013). Similar to the study, Beguy et al. (2014) have shown that married girls are less likely to experience an unintended pregnancy than unmarried girls.

A few studies have considered adolescents' psychological status such as depression and self-esteem to examine how it is related to pregnancy. Magadi and Agwandaf (2009) have found that girls with higher locus of control have a higher likelihood of being pregnant. Other studies have shown that low self-esteem is significantly related to the higher chance of pregnancy (Kanku & Mash, 2010; Lipovsek, Karim, Gutiérrez, Magnani, & Castro Gomez, 2002). A qualitative study has found that some teenagers with low self-esteem are more likely to become school drop-outs and want to have a baby (Kanku & Mash, 2010).

Religion has shown mixed results. One study has found that teenagers who are religiously affiliated have less likelihood of being pregnant (Ogland, Sunil, Bartkowski, & Xu, 2010). Some studies have argued that having no religion is positively associated with pregnancy (Haragus, 2011; McKinnon, Potter, & Garrard-Burnett, 2008). One study using data from Sub-Saharan African countries has found that being a Muslim is strongly associated with child marriage. However, their chance of giving adolescent birth is decreased (Maswikwa et al., 2015). On the other hand, according to another study conducted in Uganda, the Muslim religion is positively associated with childbirth compared to Protestant (Neal, Chandra-Mouli, & Chou, 2015). Other studies have found no significant relationship between religion and teenage motherhood (Alemayehu et al., 2010; Gurmu & Dejene, 2012).

#### 3.2.2 Self-behavior

Self-behavior refers to both active and passive behavior of adolescents such as sexual risk behaviors, substance use, and sexual/physical abuse. Many studies have revealed that sexual risk behaviors such as early sexual debut, and having many sexual partners are positively related to adolescent pregnancy (Goicolea, Wulff, Ohman, & Sebastian, 2009; Lion, Prata, & Stewart, 2009). Regarding the use of the contraceptive method, mixed results have been found in different studies. It has been found that no use or inconsistent use of contraceptive method increased the likelihood of adolescent pregnancy (e.g., Ilika & Anthony, 2004). Young people who keep using a condom are less risky to be pregnant (Toska, Cluver, Boyes, Pantelic, & Kuo, 2015). On the other hand, another study has shown the positive relationship between contraceptive uses and adolescent pregnancy (Were, 2007). However, this result does not necessarily indicate that contraceptive use increases the likelihood of teenage pregnancy. The possible reasons for the result are discussed in the Discussion section. Several studies have revealed that adolescents who engage in transactional sex for economic gains from older partners have more risks of being pregnant (e.g., Kanku & Mash, 2010; Ochiogu, Miettola, Ilika, & Vaskilampi, 2011).

Furthermore, substance use such as drinking alcohol, smoking, and drug use increases the likelihood of getting pregnant. A case-control study has found that young women who have experienced smoking have a higher chance of childbirth (Faler, Camara, Aerts, Alves, & Beria, 2013). Kanku and Mash (2010) have conducted in-depth interviews and focus group discussions with pregnant and non-pregnant girls, and women who had experienced childbirth during adolescence, and found that substance use of adolescents and their parents is a risk factor for teen pregnancy.

Sexual or physical abuse is also a risk factor related to teenage pregnancy (Christofides et al., 2014; Pallitto & Murillo, 2008). A case-control study of Ecuadorian adolescents has shown a positive association between adolescent pregnancy and abuse during childhood to adolescence (Goicolea et al., 2009). In addition to analytical studies, qualitative studies have revealed that coerced sex by boyfriend is one of the reasons that adolescents experience pregnancy (Richter & Mlambo, 2005). Girls who experienced forced first sex are more probability of getting pregnant (Jewkes, Vundule, Maforah, & Jordaan, 2001).

#### 3.2.3 Family

Family plays a critical role in human development, particularly during childhood and adolescence. Family factor refers to a family structure, parental discipline, the relationship between parent and child, communication with parents, and the education or employment status of the family. First, several studies have found that adolescents who live without both parents have a higher risk to become pregnant during adolescence (e.g., Faler et al., 2013; Goicolea et al., 2009). In regards to living with one parent, studies have shown mixed results. One study in Kenya has shown that living with only the mother is a protective factor against adolescent pregnancy (Beguy et al., 2014). However, another study in the same country has concluded that adolescents living with the father appear to be a preventing factor against adolescent pregnancy compared to living with neither parent nor only with the mother (Ngom, Magadi, & Owuor, 2003).

Regarding parental discipline, Ditsela and Van Dyk (2011) have found that adolescents who receive authoritarian or permissive parenting have a higher likelihood of getting pregnant. However, that study might have a risk of selection bias because they did not consider or control confounding factors in their descriptive study. In addition to parental discipline, girls with supportive or affectionate parents are more protected from adolescent pregnancy than their counterparts (Lipovsek et al., 2002). Moreover, communication with parents could protect adolescents from childbearing (Mturi & Moerane, 2001). Lack of discussion regarding sexual issues with parents (Khethiwe, Edwards, & Thwala, 2012; Mkhwanazi, 2010) and no parents' guidance regarding sex are risk factors associated with adolescent pregnancy (Richter & Mlambo, 2005).

Parents' level of education is also associated with teenage pregnancy. In Indonesia, when parents, especially mothers, are educated, adolescents are less likely to give birth during adolescence (Choe, Thapa, & Achmad, 2001). Gigante et al. (2004) also have found that a higher level of parental schooling is negatively associated with adolescent pregnancy. Furthermore, having family members who have experienced pregnancy before age 20 can affect adolescent pregnancy. A Brazilian study has found that adolescents are more likely to get pregnant if they have a sibling who gave birth during adolescence (Faler et al., 2013). Other studies have also presented a positive relationship between adolescent pregnancy and her mother's pregnancy before age 20 (Almeida & Aquino, 2009; Gigante et al., 2004).

#### 3.2.4 Friends

Friends have a strong influence on an adolescent's attitude and behavior during adolescence. Several studies have found that peer pressure to make adolescents sexually active or do unsafe sexual activity is one of the causes of adolescent pregnancy. In qualitative studies, teenagers have mentioned that peer or partner's pressure to engage in sexual activity and not using contraceptive method is a risk factor of adolescent pregnancy (Kanku & Mash, 2010; Richter & Mlambo, 2005). Furthermore, having time with friends who performed risk behaviours and provided information about sex and contraceptives—“sexual intercourse was an expression of love and not using condoms was an expression of fidelity (Mkhwanazi, 2010, p. 351)”—has increased the likelihood of teenage pregnancy (e.g., Mkhwanazi, 2010). However, peers do not always have negative effects on adolescents. Some studies have found that adolescents who spend time with peers are less likely to get pregnant (Lipovsek et al., 2002; Magadi & Agwandaf, 2009). Frequent dialogues about sex with boyfriends is a risk factor for adolescent pregnancy (Jewkes et al., 2001; Magadi & Agwandaf, 2009).

#### 3.2.5 School/community

The environment outside the family such as school and community can also affect adolescents. Several studies have identified external factors associated with adolescent pregnancy. School activities or community group meetings can reduce the likelihood of adolescent pregnancy (Baumgartner, Geary, Tucker, & Wedderburn, 2009). Youth forums where they discuss and learn about sex can reduce the risk of pregnancy (Salami & Ayegboyin, 2015; Were, 2007). On the other hand, when teenagers are involved in unsupervised group meetings or activities outside of school, they are more likely to be pregnant (Omar et al., 2010). In addition, lack of or low-quality sex education at school increases the risk of adolescent pregnancy (Mturi & Moerane, 2001; Richter & Mlambo, 2005). In a qualitative study, teenagers have complained that schools do not provide enough sex information (Mturi & Moerane, 2001). Furthermore, lack of access to health centers or unfriendly health care providers prevents adolescents from receiving information and contraceptive method. Such an environment can increase the risk of adolescent pregnancy (e.g., Miranda & Szwarcwald, 2007; Mkhwanazi, 2010).

Adolescent's residence and ethnicity are also associated with adolescent pregnancy, but different studies have shown mix results (e.g., Agyei et al., 2000; Choe et al., 2001). For example, adolescents living in urban Ghana are more likely to be pregnant than those in the peri-urban or rural area (Agyei et al., 2000). Another study in Kenya has found that living in a rural area reduces the likelihood of pregnancy (Magadi & Agwandaf, 2009; Maswikwa et al., 2015). However, other studies have shown the opposite result that rural residence is positively associated with teenage pregnancy (Alemayehu et al., 2010; Choe et al., 2001). Regarding ethnicity, being Oromo ethnicity in Ethiopia has been found to be a risk factor for pregnancy (Beyene et al., 2015). In Sri Lanka, Moor ethnic is a protective factor against adolescent pregnancy compared to Sinhalese and Tamils (Rajapaksa-Hewageegana, Salway, Piercy, & Samarage, 2014).

#### 3.2.6 Macro-level social, economic, and political factors

Only six studies have shown an association between adolescent pregnancy and macro-level factors including law, policies, government programs, population, and economic status of the country (e.g., Duflo, Dupas, & Kremer, 2015; Kim, Longhofer, Boyle, & Brehm, 2013). Two studies have found a significant negative relationship between the law on minimum marriage age and adolescent pregnancy (Kim et al., 2013; Maswikwa et al., 2015). Interestingly, Kim et al. (2013) have studied 114 developing countries and argued that, although the Minimum-Age-of-Marriage Laws matter, if the law is not strict or has exceptions, its effect on adolescent fertility in such countries is not different from that in countries having no minimum-age-of-marriage law. They also found that population reduction policies and international treaties worked as a protective factor related to teenage pregnancy (Kim et al., 2013). Duflo et al. (2015) have evaluated the impact of the government's programs and found that education subsidy programs reduced girls' pregnancy whereas HIV education programs had no significant impact on their pregnancy.

## 4 Discussion

Adolescence is an important period when many changes occur partly due to social and environmental changes around adolescents. Pregnancy in adolescence is caused by the surrounding micro- and macro-environments and by the interaction with various determinants. A total of 67 studies focused on factors or predictors associated with teenage pregnancy in developing countries. Compared to previous systematic reviews, this study covered a broader range of studies to identify a more comprehensive set of issues related to the determinants of adolescent pregnancy. Studies selected for this systematic review utilized various research methods, and covered many low- and middle-income countries around the world. The age range of the sample population varied across the studies. Many studies included adolescents aged from 10 to 19 years, but the most commonly used age group was between 15 and 19 years old. Some studies included young people aged 20–24 years or adults aged over 25 years for one of the following reasons: 1) to compare adolescents and adults; 2) to examine adults who gave birth during their adolescence; and 3) to compare both groups and separate the result of adolescents from that of adults.

Similar to previous systematic reviews, there are consistent results for the relationship between adolescent pregnancy and its causal factors. Older age, lower level of education, poor economic status, and early marriage can increase the hazards of getting pregnant (Acharya, Bhattarai, Poobalan, Teijlingen, & Chapman, 2014; Pradhan et al., 2015). Sexual risk behaviors are also risk factors in most reviews (e.g., Mmari & Blum, 2009). However, unlike our study, previous systematic reviews rarely showed psychological factors or sexual/physical abuse as factors for adolescent pregnancy. As mentioned earlier, scholars have found that adolescents with depressive symptoms or low self-esteem are more likely to become pregnant than their counterparts. This is in line with other studies showing that adolescents with low self-esteem are more likely to engage in risky sexual behaviors and less likely to use contraceptives (e.g., Miller, Forehand, & Kotchick, 2000). In addition to the risk factors found in the previous reviews such as family disruption and having a family member who gave birth in adolescence, we also found that lack of parental support, less communication with parents, and low level of parental education make adolescents more vulnerable to pregnancy. Peer pressure and inaccurate information from friends put adolescents into risks of unwanted pregnancy. Lack of sex education at school or low quality and limited access to the health center can lead adolescents to engage in risky sexual behaviors (e.g., Mturi & Moerane, 2001).

Several factors have mixed and complicated associations with teenage pregnancy. Religion has shown either a negative or a positive association with adolescent pregnancy in different studies, in line with a previous systematic review (Pradhan et al., 2015). This study has also found that religion is not significantly related to adolescent pregnancy. It is supported by an argument of Were (2007) that religion itself might not affect adolescent pregnancy, but religious meeting with adequate information for adolescents would affect their health. Low use of contraceptive method can increase the likelihood of being pregnant. However, the opposite result has been reported that adolescents who continuously used contraceptive method are more likely to be pregnant (Were, 2007). The author has presented several reasons for the results: the study has not considered sub-factors related to contraceptive use; adolescents are likely to start using the method after experiencing pregnancy; and they might misuse the method (Were, 2007, p. 336). This suggests that further study needs to take more research on contraception. Studies have shown mixed results regarding living areas. Some studies have shown that living in a rural area is a protective factor while others have shown that living in a rural area is a risk factor for teen pregnancy. Moreover, several factors including substance use, religion, orphanhood, and income are not significantly associated with adolescent pregnancy in several studies. Inconsistent results imply that certain factors might have varied associations with pregnancy due to different contexts. Thus, we need to consider a specific context or detail background of adolescents to answer complicated and inconsistent relationships between various factors and adolescent pregnancy.

Although most studies have considered several factors affecting pregnancy, few studies have examined complex interactions among these factors. For instance, when we think of the relationship between low economic status and adolescent pregnancy, several factors might affect the relationship between the two factors. An adolescent who is suffering from poverty might have low self-esteem due to his/her economic condition or live in conditions vulnerable to sexual abuse or violence. His/her friends might influence him/her to engage in sex with an older rich person for economic gains. Thus, it is crucial to examine the complex interaction of several factors that affect teenage pregnancy.

Furthermore, author's characterization of teenage pregnancy matters–i.e., Results of studies appear to be different depending on how the author identifies teenage pregnancy (e.g., desired, unwanted, or unplanned pregnancy). For example, although older adolescents are more likely to be pregnant than younger ones, younger participants reported more unwanted or unintended pregnancy than older ones (Amoran, 2012). However, most studies did not distinguish factors among desired, unwanted, and unplanned pregnancy. To enhance understanding of adolescent pregnancy, it is critical to assess pregnancy desire among adolescents (Christofides et al., 2014).

Most studies that we reviewed in this study were cross-sectional studies or descriptive survey. Cross-sectional studies have some limitations since it is difficult to find causality or dynamic impact across time. In addition, descriptive studies do not consider confounding factors that might affect the relationship between factors and pregnancy. More studies that collect and analyze longitudinal data are needed to reduce such limitations. Furthermore, mixed-method study with both quantitative and qualitative methods can compensate methodological weaknesses of previous studies by increasing reliability as well as showing complexity among factors. Through mixed-method study, quantitative research can show a clear association between factors and pregnancy while qualitative study can provide detail mechanisms or processes within associations. Although this study aimed to include all literature regarding factors related to adolescent pregnancy, our search strategy limited to English-language articles and using four databases. Some related articles might not be included in the study. In addition, we synthesized the articles and simplified the results of each study. There might be a reporting bias by revealing selective information. Finally, the studies we reviewed in the study have different study sample, methods, and analyses. Furthermore, the quality of the studies varies. Thus, we need to interpret and generalize the findings cautiously.

## 5 Conclusion

Many studies have examined factors associated with adolescent pregnancy in low- and middle-income countries. From micro-to macro-level studies, several factors are found to be associated with adolescents' health, especially their pregnancy. This study provides a comprehensive understanding of risk factors and protective factors for teenage pregnancy. This study also attempted to uncover the complex relationship between factors and teenage pregnancy. Results of this systematic review suggest that future research needs to examine interactions among factors and study the mediating or moderating effect among various factors. Policy makers and program officers need to plan pregnancy prevention programs by considering individual, school or community, and country level of risk factors for adolescent pregnancy. Also, the specific context and background of adolescents and their health need to be taken into account.

## Funding

This research was supported by Bill & Melinda Gates Foundation, United States, Grant No. OPP1055880 and No. OPP1150374, and by Ewha Womans University, Republic of Korea, Grant No. 1-2015-2036-001-1.
