# Supplementary material for: Comprehensive understanding of risk and protective factors related to adolescent pregnancy in low- and middle-income countries: A systematic review
Source: J Adolesc. 2018 Dec;69:180–8. doi: 10.1016/j.adolescence.2018.10.007 (PMC6284104; doi:10.1016/j.adolescence.2018.10.007)
Supplement: Multimedia component 1 [file mmc1.docx]

**Appendix.** Characteristics of the included studies

| Authors (Year) | Sample Size & Characteristics | Region | Analytic Methods | Outcome | Key Findings^a^ |
| --- | --- | --- | --- | --- | --- |
| ***Case-control study*** | | | | | |
| **Baumgartner et al. (2009)** | Cases (n=250): pregnant girls aged 15-17; Controls (n=500): sexually experienced but never-pregnant girls (neighborhood-matched) | Jamaica | Bivariate, multivariate(Conditional logistic regression) | Adolescent pregnancy | Currently in stable relationship(+), lower self-esteem(+), first sexual partner was 5 years older(+), believing that contraception is woman’s responsibility(+), experience sexual violence(-), being involved in community groups(-), think important to protect self against pregnancy(-) |
| **Faler et al. (2013)** | Cases (n=431): girls aged 14-16 who gave birth in 2009; Controls (n=862): Never-pregnant girls | Porto Alegre, Rio Grande do Sul State, Brazil, | Bivariate, multivariate logistic regression | Adolescent motherhood | Lower economic class(+), absence of both parents from 10 to 14 years of age(+), having taken care of other children(+), siblings who gave a birth in their adolescence(+), having tried smoking(+), having arrived home intoxicated(+) |
| **Gigante et al. (2004)** | Cases (n=420): girls included in the 1982 cohort study; Controls (n=408): girls who were located in the 2001 follow-up and had not given birth before 31 Mar 2001 | Pelotas, Rio Grande do Sul, Brazil | Multivariate(Unconditional logistic regression) | Adolescent childbearing | Higher levels of family income(-), of maternal schooling(-), and of paternal schooling(-), maternal religious belief (traditional)(+), mother gave birth during adolescence(+), had siblings of different fathers(+), school failure up to 4^th^ grade(+), younger age at first intercourse(+) |
| **Goicolea et al. (2009)** | Cases (n=140): pregnant girls aged 10-19 or had been pregnant for the first time during the previous 2 years; Controls (n=262): never-pregnant girls | Orellana, Ecuador | Bivariate, multivariate(Conditional logistic regression) | Adolescent pregnancy | early sexual debut(+), very poor household(+), experience sexual abuse during childhood-adolescence(+), absence of both parents in some periods of adolescence(+), non-use of contraception during first sex(+) |
| **Jewkes et al. (2001)** | Cases (n=191): African girls under 19 years in 1995; Controls (n=353): never-pregnant girls (age and school matched) | Cape Town, South Africa | Multivariate(Conditional logistic regression) | Adolescent pregnancy | frequent sex without injectable contraceptives(+), forced sexual initiation(+), lack of TV ownership(+), having a larger household size(+), not living in a brick house(+), not living with a father(+), talking openly about sex with boyfriend(+), and beliefs that most friends are pregnant(+) |
| **Omar et al. (2010)** | Cases (n=102): girls aged 10-19, delivered at one of two hospitals; Controls (n=102): women aged 20-35, delivered (same day and place) but no pregnancy in their adolescence | Kuala Lumpur, Malaysia | Bivariate(Fisher exact test) | Adolescent pregnancy | Lower education level(+), lower economic status(+), unemployment(+), unmarried(+), substance use(+), being raised by single parent(+), unsupervised activities after school (+), less participated extra activities at school(+) |
| **Moron-Duarte, Latorre and Tovar (2014)** | Cases (n=272): pregnant girls aged 14–19; Controls (n=544): never-pregnant girls (randomly-selected) | Bogota, Colombia | Bivariate, multivariate(Conditional logistic regression) | Adolescent pregnancy | going public school(+), not living with both parents(+), sibling with history of adolescent pregnancy(+), had first sex age 12 or younger(+), having a self-reported low or average level of contraceptive knowledge(+), previous pregnancy(+) |
| ***Cohort study*** | | | | | |
| **Beguy, Mumah, and Gottschalk (2014)** | Young women aged 15–22 (n=849) in two slums (3-year prospective cohort study, 2007-2010) | Nairobi, Kenya | Multivariate(Cox and logistic regression) | Unintended pregnancy | being in school(-), being married(-), had first sex b/w ages 15-17(+), living with father or both parents(+), initiate sex before 18*sexual debut b/w ages 15-17(+) |
| **Christofides et al. (2014)** | Girls aged 15-18 (n=819) at baseline from 70 villages and residential areas near Mthatha (2 years of follow-up) | Eastern Cape, South Africa | Multivariate(Polytomous regression) | Unplanned and unwanted pregnancy | hormonal contraception(-, for unplanned pregnancy), physical abuse(+, for unwanted pregnancy), lower socioeconomic status(+, for both), mutual main partners(+, for both), having pregnancy prior to baseline(-, for unwanted) |
| **Rosenberg et al. (2015)** | Girls aged 12–18 (n=15,457) (longitudinal demographic surveillance data, 2000-2011) | Agincourt sub-district, South Africa | Multivariate(Cox proportional hazard) | Adolescent pregnancy | enrolled in school(-), school term(-, vs. vacation) |

*(Continued)*

| Authors (Year) | Sample Size & Characteristics | Region | Analytic Methods | Outcome | Key Findings^a^ |
| --- | --- | --- | --- | --- | --- |
| *Analytical cross-sectional study* | | | | | |
| Alemayehu, Haider, and Habte (2010) | Girls aged 15-19 (n=3,266) (Ethiopian Demographic Health Survey 2005) | 9 regions of Ethiopia | Multivariate logistic regression | Adolescent fertility | No or primary education(+, vs. secondary and above), living in rural or other urban area outside of Addis Ababa(+), current age 18-19(+, vs. age 15-17), currently not working(+) |
| Almeida and Aquino (2009) | Youth aged 20-24 (n=2,701) (A Multicenter Study on Young Persons, Sexuality and Reproduction in Brazil, 2002) | Porto Alegre, Rio de Janeiro, Salvador, Brazil | Multivariate logistic regression | Adolescent pregnancy | whose mother gave a birth before aged 20(+) (education level eliminates this association for female) |
| Amoran (2012) | Pregnant women aged 14-40 (n=225) attending primary health care within a 2 months period (Comparative study: adolescents vs. adults) | Ogun state, Southwestern part of Nigeria | Univariate, bivariate | Adolescent pregnancy | lower level of education(+, vs. post-secondary education), not know how to correctly use condom(+), low social class(+), Christianity(-, vs. Islam), being a student(+), having a white collar job(-), Hausas ethnicity(+) |
| Beyene et al. (2015) | Female adolescents (n=770) visited a hospital (mean age: 16.86±1.95 years) | Assosa, Ethiopia | Bivariate, multivariate | Adolescent pregnancy | being young(-), unmarried(-), lower family income(-), housemaid(+), being Oromo ethnicity(+), non-use of family planning(+) |
| Brahmbhatt et al. (2014) | Adolescents aged 15-19 from Baltimore (n=456), Johannesburg (n=496), Ibadan (n=449), New Delhi (n=500), and Shanghai (n=438) living in disadvantaged urban areas | USA, South Africa, Nigeria, India | Multivariate logistic regression | Adolescent pregnancy | [South Africa] [Female] being raised by a single parent or by other(+), unstable housing(+), had first sex at age 15 or younger(+), neighborhood violence(+), fear of being robbed or attacked(+), being in school(-), using condom at first sex(-), better perceived physical environment(-)  [Male] age(+), early sexual debut(+), ever engaging in transactional sex(+), currently in school(-), perceived fear of being robbed or attacked(-), better perceived physical environment(-) |
| Calvert et al. (2013) | Young adults aged 15-30 (n=13,814) (MEMA kwa Vijiana Trial Long-term Evaluation Survey) | Mwanza, Tanzania | Multivariate(Conditional logistic regression) | Unplanned pregnancy | [women aged 15-24 years] age(+), lower educational level(+), currently unmarried(+), younger age at first sex(+), increasing number of lifetime partners(+) |
| Chiavegatto and Kawachi (2015) | Births (n=1,247,145) from girls aged 15-19 in 2000-2010, 5,565 municipalities | Brazil | Multivariate | Adolescent fertility | Income inequality (+) |
| Choe, Thapa, and Achmad (2001) | Women (n=3,978) and men (n=4,106) aged 15–24/ Indonesia; Women (n=1,445) and men (n=1,379) in urban and women (n=2,730) and men (n=2,423) in rural areas aged 14-22/ Nepal | Indonesia, Nepal | Multivariate | Adolescent childbearing | [Both countries] education(-), rural(+); [Indonesia] parent’s education(-) (particularly mother’s); [Nepal] Terai region(+, vs. hill region) |
| Choe, Thapa, and Mishra (2005) | Youths in urban (n=2,800), rural (n=5,075) aged 14–22 (2000 Nepal Adolescent and Young Adult Survey) | Nepal | Multivariate(proportional hazard, logistic regression) | Early motherhood | [Urban female] year of birth(-), father’s higher level of education (-, vs. none), rural birthplace(+, vs. urban), higher level of education(-, vs. none), living in Pokhara, Biratnagar, Birgunj area(+, vs. Kathmandu), Newar or Gurung/Magar/Tamang/Rai/Limbu ethnicity(-, vs. others)  [Rural female] year of birth(-), rural birthplace(+, vs. urban), higher level of education(-, vs. none), more developed district(-, vs. less developed), Gurung/Magar/Tamang/Rai/Limbu ethnicity(-, vs. others) |
| Gupta and Mahy (2003) | Youth aged 18-24 (Demographic and  Health Surveys, 1987-1999) | 8 Sub-Saharan African countries | Multiple logistic regressions | First birth in adolescence | girls’ education from secondary level(-) |
| Gurmu and Dejene (2012) | Women aged 20-49 (n=10,818) (2005 Ethiopian Demographic and Health Survey) | Ethiopia | Multilevel logistic regression | Adolescent motherhood | sexual experience before age 15(+), married before 15(+), earlier maternal age cohort(+), higher level of education(-), exposure to media(-),non-agricultural worker(-), urban residence(-), some regions(+) |

*(Continued)*

| Authors (Year) | Sample Size & Characteristics | Region | Analytic Methods | Outcome | Key Findings^a^ |
| --- | --- | --- | --- | --- | --- |
| *Analytical cross-sectional study* | | | | | |
| Haragus (2011) | Women aged 45 or younger (Generations and Gender Survey for Romania 2005) | Romania | Multivariate(event history models) | Teenage birth | [non-marital teenage birth] more than 3 siblings(+), mother’s education(-), Roma ethnicity(+), not being religious(+), being enrolled in school(-), medium level of education(-, vs. low educational attainment), had already left parental home(+), fall of socialist regime(+) |
| Kim et al. (2013) | 1,757 observations from data for 114 poor- and middle-income countries, 1989-2007 | 114 developing countries | Multivariate(panel regression) | Adolescent fertility | strict laws setting the minimum age of marriage at 18(-), female secondary enrollment(-), population reduction policies(-), international treaties(-) |
| Lanjakornsiripan et al. (2015) | Pregnant girls aged 15-19 attending hospital and association | Khon Kaen, Thailand | Univariate, multivariate(Logistic regression) | Unintended pregnancy | In school (+, vs. completed education), age 16 or younger (+, vs. age 17 or older) |
| Lion, Prata, and Stewart (2009) | Girls aged 15-19 (n=3,142) (2001 Nicaragua Demographic and Health Survey) | Nicaragua | Bivariate, multivariate(Cox proportional hazard) | Earlier first birth | Had first sex before age 15(+), had first sex at age 16 or later(-) |
| Magadi and Agwandaf (2009) | Girls aged 12–19 (n=1,247) (The Adolescent Safe Motherhood Survey 2002) | South Nyanza, Kenya | Bivariate, multivariate(Cox regression) | First pregnancy | high household socioeconomic status(-), secondary or higher level of education(-), discuss sexual matters with boyfriends(+), discuss sexual matters with peers(-), rural residence (-), higher internal locus of control(+), having strong gender bias(+) |
| Maswikwa et al. (2015) | Women aged 15–26 (Demographic and Health Surveys, and Child Marriage Data) | 12 Sub-Saharan African countries | Multivariate logistic regressions | Adolescent childbearing | consistent law(-), child marriage(+), poverty(+), not belonging to religion(+), higher educational attain(-), rural residence(-), affiliation with Islam(-), traditional religion(-) |
| McHunu et al. (2012) | Youth aged 18-24 (n=3,123) | 4 provinces, South Africa | Bivariate, multivariate(Unconditional logistic regression) | Adolescent pregnancy | [Female] being employed or unemployed, greater poverty, having higher sexually permissive attitudes and scoring higher on the contraceptive or the condom use index (+), [Male] wanting the pregnancy and having a sense of the future (+) |
| McKinnon, Potter, and Garrard-Burnett (2008) | Adolescents aged 15-17 (n=29,021) (2000 Brazil Census) | Rio de Janeiro, Brazil | Multivariate(Logistic regression) | Adolescent fertility | Protestants(-, vs. Catholics), no religion(+, vs. Catholics), education(-), race: black and brown(+), migrant status(+), age above 15(+), higher socioeconomic status(-) |
| Miranda and Szwarcwald (2007) | Girls (n=464) aged 15-19 | Victoria, Brazil | Bivariate, multivariate(Logistic regression) | Adolescent pregnancy | Not living with parents(+), not having access to health service(+), had first sex younger than age 15(+), less than 9 years of schooling(+) |
| Neal, Chandra-Mouli, and Chou (2015) | Women aged 20–24 who had their first birth at age less than 16, 16-17 and 18-19 in Kenya, Uganda, and Tanzania | Kenya, Uganda, and Tanzania | Bivariate, multinomial logistic regression | Adolescent motherhood | [All countries] poverty(+), lack of education/literacy(+), marked differences by region, religion and urban/rural residence [Uganda] Muslim religion (+, first birth aged before 20, vs. Protestant) [Kenya] Catholic (+, first birth aged before 16, vs. Protestant) |
| Ngom, Magadi, and Owuor (2003) | Unmarried girls aged 12-19 (n=788) living in slums (Nairobi Slums Cross-Sectional  Survey 2000 and 1998 Kenya  Demographic and Health Survey) | Nairobi, Kenya | Logistic regression | Unwanted pregnancy | father is present in the household (-, vs. when neither parent or only the mother) |
| Ogland et al. (2010) | Unmarried girls aged 15-19 (n=2,364) (2006 National Demographic and Health Survey) | Brazil | Logistic regression | Adolescent childbearing | religiously affiliated(-), Pentecostal affiliation(-), attend worship services frequently(-), greater wealth(-), higher educational achievement(-), working for pay(-) |

*(Continued)*

| Authors (Year) | Sample Size & Characteristics | Region | Analytic Methods | Outcome | Key Findings^a^ |
| --- | --- | --- | --- | --- | --- |
| *Analytical cross-sectional study (cont.)* | | | | | |
| Okigbo and Speizer (2015) | Unmarried women (n=2,020) aged 15-24 currently not living with male partner | five urban areas in Kenya | Cox proportional hazard regression and logistic regression | First pregnancy and adolescent pregnancy | secondary or higher education(-), richest wealth(-), used modern contraception at first sex(-), living in Kisumu(+, vs. Nairobi), early sexual debut(+) [First pregnancy] living in Kisumu and Kakamega(+, vs. Nairobi), living in a large household(+), living in a mother-headed household(+, father-headed), knowing at least one contraceptive method(+), high level of family planning misperceptions(+), early sexual debut(+), secondary or higher education(-), living in Mombasa(-) |
| Palermo and Peterman (2009) | Girls aged 15-17 (Demographic and Health Surveys) | 10 sub-Saharan African countries | Bivariate, logistic regression | Adolescent pregnancy | orphanhood(little association) |
| Pallitto and Murillo (2008) | Women aged 15-24 (n=3,753) (compared to the group of women aged 25-49) | El Salvador | Multiple logistic regression | Adolescent pregnancy | sexual abuse(+), physical abuse(+), any abuse(+) during childhood, intimate partner abuse(+) |
| Rajapaksa-Hewageegana et al. (2014) | Pregnant girls (n=450) and male partners of pregnant women (n=150), both aged less than 20 | Badulla District, Sri Lanka | Bivariate, multivariate logistic regression | Unplanned pregnancy | Moor ethnicity(-, vs. Sinhalese, Tamils), not wanting the first intercourse(+), not being happy as a teen(+) |
| Roza and Martinez (2015) | 853 Municipalities, in 2010 (the Brazilian Live Birth Information System) | Minas Gerais, Brazil | Multivariate | Live birth to adolescents | low population density(+), low human development index(+), percent of families supported by the Bolsa Familia Program(+), low Municipal development index(+), low social responsibility index(+) |
| Sahoo (2011) | Women aged 15-19 (District Level Household and Facility Survey 3) 2007-2008 | India | Bivariate, multivariate(multiple classification analysis) | Adolescent fertility | scheduled tribe(+), educational level(-), living in urban(+), age(+) |
| Sintonen, Bonilla-Carrion, and Ashorn (2013) | Costa Rican and Nicaraguan-born women aged 12-19 (n=318,379) (IX National Population and Housing Census), 2000 | Costa Rica | Univariate, bivariate, Multivariate logistic regression | Adolescent childbearing | Nicaraguan-born(+), age(+), low educational attainment(+), urban residence(+), poverty(+), union(+) |
| Sriprasert et al. (2015) | Pregnant women aged 15-24 (n=250) at prenatal clinic | Chiang Mai, Thailand | Univariate, stepwise logistic regression | Unintended pregnancy | being students(+, vs. completed education), women aged 20 years or younger(+, vs. older than 20 years), partner was a similar age(+) |
| Stark et al. (2015) | Adolescents aged 13-19 (n=530) | 2 districts in Sierra Leone | Bivariable and multivariable logistic regressions | Recent pregnancy | Living arrangement (family structure) (x), orphanhood (x) |
| Toska et al. (2015) | Sexually-active girls aged 10-19 (n=447) | 3 provinces in South Africa | Univariate and multivariate logistic regressions | Adolescent pregnancy | Consistent condom use(-), school enrolment(-), engaging in age-disparate sex(+), long-term school absences(+) |
| Vazquez-Nava et al. (2014) | Girls aged 13-19 (n=3,130) in urban area | northeastern Mexico | Univariate, logistic regression models | Unplanned pregnancy | having an employed mother(+), being an active smoker(+), having girlfriends with health-risk habits/behaviors(+) |
| Wei and Yu (2013) | Pregnant women (n=895) and non-pregnant women (n=611) aged 15-24 in youth clinics | Beijing, Guangzhou, Jinan in China | Multiple logistic regression | Adolescent pregnancy | adverse childhood experience(+), parents were separated or divorced(+) |
| *Panel study* | | | | | |
| Were (2007) | Adolescents aged 10–19 (female n=203, male n=55) | Busia District in Kenya | Logistic regression, qualitative (in-depth interviews and focus group discussions) | Adolescent pregnancy | education level(-), Church forums that discuss about sex/family life(-), age(+), use of contraceptives(+), [view of adolescents] peer pressure(+), social environment-related factors(+), lack of parental guidance and counselling(+) |

*(Continued)*

| Authors (Year) | Sample Size & Characteristics | Region | Analytic Methods | Outcome | Key Findings^a^ |
| --- | --- | --- | --- | --- | --- |
| *Randomized controlled trial study* | | | | | |
| Handa et al. (2015) | Females aged 12-24 (n=1,549) | Kenya | Probit regressions | Adolescent pregnancy | Cash Transfer program(-) through increasing female school enrolment, financial stability, and delaying age at first sex |
| Duflo, Dupas, and Kremer (2015) | Adolescents aged 13-20, 328 schools (Control=82; Education Subsidy=83; HIV Education=83; Joint=80) | Western province, Kenya | Ordinary Least Squares regression | Adolescent pregnancy | Education subsidy program(-), joint program(-) |
| *Mixed method study* | | | | | |
| Lipovsek et al. (2002) | Cases (n=95) girls aged 13-19; controls (n=95) in a peri-urban area | La Paz, Bolivia | Bivariate, conditional logistic regression, FGDs | Adolescent pregnancy | [Quantitative] fighting in your home(+), parents are affectionate(-), parents are supportive(-), ever talk with partner about pregnancy(+), get together with friends often(-), self-esteem(-) [Qualitative] partner(peer) pressure to have sex and not to use contraceptives, difficult to obtain accurate info on reproduction and contraception, learned biological aspects of sex in school, where info about sex or contra could be obtain are listed in order as friends, media, school, and family (family rarely gives info), girls cannot demand the use of contraceptives |
| Marteleto, Lam, and Ranchhod (2008) | Young people (n=3,916) (Cape Area Panel Study) | metropolitan Cape Town, South Africa | Probit regressions | Adolescent pregnancy | being enrolled(-), colored(+), household shock(+, girls only) |
| *Descriptive study (Qualitative study & cross-sectional survey)* | | | | | |
| Ilika and Anthony (2004) | Unmarried pregnant girls (n=136) attending a hospital | Ozubulu, rural, South-east Nigeria | Descriptive statistics, in-depth interview | Unintended pregnancy | multiple partners(+), sex for economic reasons and exchanged sex for money or gifts(+), not using condoms(+) |
| Okereke (2010) | In-school and out-of-school girls aged 10-19 (n=540), health-care service providers | Owerri, Nigeria | Descriptive statistics, FGDs, in-depth interviews | Unintended pregnancy | Concerns about the side-effects of contraceptive methods(+), rarely go to health centers(+), Catholics would refrain from contraceptive use(+), unwilling to buy contraceptives at the centers or pharmacy(+), inappropriate channels to dissemination information about pregnancy(+), transactional sex(+) |
| Salami and Ayegboyin (2015) | Pregnant teenagers (n=163) (boy and girls), teen parents (n=91) and adult parents (n=23) who were once teen parents | Ogbomosho, Nigeria | Descriptive statistics, FGDs | Adolescent pregnancy | lack of knowledge about puberty, improper care from the family or lack of parental care, financial problems, sexual harassment, keeping bad company with peers, low knowledge of sexual education and protection, and disobedience to parents(+) good parental care and counselling, youth forums on sex education, and free education by government (-) |
| *Cross-sectional survey* | | | | | |
| Agyei et al. (2000) | Unmarried females (n=829), males (n=953) aged 15-24 (Adolescent Fertility Survey, Ghana 1996) | Greater Accra and Eastern Regions, Ghana | Descriptive statistics, bivariate analyses | Adolescent pregnancy | urban(+, vs. peri-urban, rural areas), age(+) |
| Almeida, Aquino, and Barros (2006) | Young people aged 20-24 (n=3,042) (A Multi-center Study on Youth, Sexuality,  and Reproduction in Brazil) | Salvador, Rio de Janeiro, and Porto Alegre in Brazil | Univariate and bivariate analyses | Adolescent pregnancy | irregular school records(+), incomplete elementary school(+), temporary dropout especially for girls(+) |
| Ditsela and Van Dyk (2011) | Adolescents and young adults (n=121) | Saldanha area, West Coast of South Africa | Descriptive statistics | Adolescent pregnancy | authoritarian parenting(+), permissive parenting style(+), low self-esteem(+), low locus of control(+), permissive, authoritative, authoritarian parenting styles of single fathers(+) |

*(Continued)*

| Authors (Year) | Sample Size & Characteristics | Region | Analytic Methods | Outcome | Key Findings^a^ |
| --- | --- | --- | --- | --- | --- |
| *Cross-sectional survey* | | | | | |
| Gomes et al. (2008) | Pregnant or puerpera adolescents (n=278), aged 15–19 (compared to older groups) | Teresina, Brazil | Descriptive analyses | Adolescent pregnancy | not in school(+), low schooling of mother(+) |
| Manzini (2001) | In-school and out-of-school girls (n=796) aged 14-22 | KwaZulu Natal, South Africa | Descriptive statistics | Adolescent pregnancy | [among pregnant girls] age(+), African(+), no longer in school(+), not completed secondary education(+), currently not schooling but had secondary education(+) |
| Mothiba and Maputle (2012) | Pregnant girls (n=103) aged 13-19 attending antenatal care in Capricorn District | Limpopo Province, South Africa | Descriptive statistics | Adolescent pregnancy | early sexual debut(+), older partners(+), not using contraceptive method(+), depending on a single parents' income > social grant > pension fund of grandparents(+) |
| Mturi and Moerane (2001) | Women aged 15-24 (n=1,513) (Lesotho Safe Motherhood Initiative Survey, 1995) | Lesotho | Descriptive statistics | Premarital pregnancy | socio-cultural modernization including attending schools away from home(+), practices such as dating (undermines traditional culture against premarital sex)(+), lack of communication between parents and children(+), children are no longer as closely supervised(+), lack of sex education from schools(+) |
| Ochiogu et al. (2011) | Students aged 14-17 (n=1,234), teachers (n=46) in 5 secondary schools | Southeastern Nigeria | Descriptive statistics | Unintended pregnancy | [Teachers] financial need and poor parental support(+), marital promise or peer pressure to get married(+) [Students] sex in exchange for money(+), marital promise or peer pressure to get married(+), ignorance(+) |
| Sant'Anna et al. (2006) | Pregnant girls (n=152) attending dept. of Pediatrics | Sao Paulo, Brazil | Descriptive statistics | Adolescent pregnancy | not use any contraceptive method(+), from unstructured families(+), high dropout rate(+), early age at first intercourse(+) |
| *Qualitative study* | | | | | |
| Kanku and Mash (2010) | Women aged 19-25 (n=10) who had given birth as teen, non-pregnant girls (n=14), pregnant girls (n=13) aged 16-19 | Taung, rural, South Africa | In-depth interviews, Focus group discussions (FGDs) | Adolescent pregnancy | poverty, child support grant, transgenerational sex, substance use of teen or her parents, lack of entertainment and social infrastructure, pressure from boyfriends and social network, parent's perception that marriage is better than HIV, poor sexual negotiation skills, need to prove one's fertility, sexual coercion, low self-esteem |
| Khethiwe, Edwards, and Thwala (2012) | Young Xhosa women (n=10) who had unwanted pregnancies living in disadvantaged area | Eastern Cape, South Africa | FGDs, inductive analysis | Unwanted pregnancy | Parents do not actively discuss sexual relationships and contraception, parents are too strict, stigma on contraception, careless and ignorance towards contraception, transactional sex due to poverty |
| Mkhwanazi (2010) | Youth aged 16-25 who gave a birth or women had given birth during adolescence (five-year study) | Western Cape, South Africa | Ethnographic, interviews | Adolescent pregnancy | lack of discussion about sex with parents, only girls receive formal instruction on their behavior, get sex information from peers (promote sex), having sex and not using condom means love and faith for peers, lack of info. on contraception, barriers to get access to contraception (fear of going to clinic, rude nurses) |
| Richter and Mlambo (2005) | Girls (n=22) and boys (n=10) aged 13-19, attended clinic or youth center | Limpopo Province, South Africa | Face-to-face interviews, Tesch’s method of data analysis | Adolescent pregnancy | lack of knowledge about contraception, misconceptions on contraceptives, boys are reluctant to use condom due to unsatisfied feeling, poor knowledge and ignorance of conception, poor quality of sex education, no sex education at home, sexual coercion by boyfriend, boyfriend's pressure, boyfriend’s refusal to use condom, peer influence, parental influences to marry early, lack of access to health services |
| Sekiwunga and Whyte (2009) | Girls aged 13-15 or 16-20, boys aged 15-20, mothers of teens, community leaders | Busia District, rural, Uganda | FGD, interviews, thematic analysis | Adolescent pregnancy | [Views of adolescents, leaders] parents or guardians do not care and control their daughters, parents are not strict or too harsh, parent's mistreatment, parents fail to provide basic needs, no info. and guidance on sexuality, government’s safe sex programs did not target adolescents, parents pressure to marry (bride wealth), parents think that girls' education has low value, defilement law (sex under 18 is criminal) is not applied [of mothers] girls’ misbehaving, peer pressure, community members interruption on parent’s discipline, grandparents' pressure to marry early |

*Note.* In results of univariate, bivariate, and multivariate analyses, (+) refers to the positive association and (-) refers to the negative association between dependent (outcome) and independent variables (key finding). In case of descriptive and qualitative study, (+) refers to risk factors and (-) refers to protective factors for outcome

^a^This is a simple summary of results. Findings include factors that are significantly associated with the outcome of each study.
